# Nasal NK/T-Cell Lymphoma. A Comparative Analysis of a Mexican Population with the Other Populations of Latin-America

**DOI:** 10.4084/MJHID.2015.052

**Published:** 2015-09-01

**Authors:** Agustin Avilés

**Affiliations:** Oncology Research Unit. Oncology Hospital National Medical Center, IMSS, México DF, Mexico.

## Abstract

Nasal natural killer/T-cell lymphoma (nasal NKTCL), is a rare presentation of extranodal lymphoma in North-America and Europe, but in some countries, as China and Korea, nasal NKTCL occurred in 20 to 46 % of T-cell lymphomas. Some studies, analyzing the incidence in Latin-America, observed some differences between the various populations. However, this comparison included Argentina and Chile, Peru, and other Latin-America but not the Mexico. Thus, we performed a retrospective analysis of the patients diagnosed and treated as nasal, NKTCL, in our institution that is an academic tertiary national reference hospital of Mexico.

From 1988 to 2014, we diagnosed and treated 14,816 cases of non-Hodgkin’s lymphoma, 10,957 (73%) were of B-cell histology and 3822 (26%) were of T-cell histology. Nasal, NKTCL, was the most frequent of the T-cell histology: 40%. We compared our results with those of other countries and observed that nasal, NKTCL have a small number of cases in North-America, and in some countries of Latin-America, as Argentina, Brazil, and Chile. However, the number of NKTCL cases found in Mexico was similar to that found in Guatemala and Peru, and also in China and Korea. Our study suggests that this neoplasm could have a racial basis, but environmental factors should also be considered.

## Introduction

Nasal NK/T-cell lymphoma (NKTCL), is a rare presentation of malignant lymphoma with protean clinical features, characterized by destruction of the upper respiratory tract, in particular of the nasal cavity, nasal and paranasal sinuses, and hard palate.^1^

It is more common in Asia, and in some countries of Latin America, as Guatemala and Peru than in Western countries.^1^

Mexico is a country that geographically and politically is part of North-America, but with racial differences, that can reflect the differences in some neoplasm, specifically NK/TCL. Although some environmental factors have to be considered as part of these differences, until now, the racial differences appear mostly to be the cause of the high proportion of these special setting of patients in Mexico.

Thus, we performed a retrospective analysis of patients with NK/TCL, which were diagnosed and treated at our Hospital. The Oncology Hospital at National Medical, is a tertiary national reference center for patients with cancer, in the Mexican Institute of Social Security. Although our institution has a national coverage with 53,000,000 of people; we cannot considered these study as a national study. Subsequently, we searched for reports of NKTCL, in other countries. Some of these patients have been previously reported.^2^–^7^

## Patients and Methods

We, while searching clinical records of the patients from 1988 to 2014 with a diagnosis of Non-Hodgkin lymphoma, separated patients with a confirmed diagnosis, according to the criteria of the World Health Organization. From 2009 to 2012, our Pathology Department performed a revision of all T-cell lymphomas, which were reclassified according to the World Health Organization.

Entry criteria were as follow: age > 18 years without upper limit; no gender differences; at the immunohistochemical studies, all lymphomas were CD2+, cytoplasm CD3epsilon +, CD56+, and expressing perforin enzyme B, TIA. Evidence of Epstein-Barr virus was shown by in situ hybridization. Prognostic factors were evaluated according to the International Prognostic Index (IPI) and the Korean proposal. Staging studies were performed as previously mentioned;^7^ positive emission tomography (PET) was added since available in our institution (2008).

Treatment was made as previously reported,^5^ and was based on the administration of combined therapy, chemotherapy and radiotherapy, most cases as the “sandwich “ technique, that at present is considered the treatment of choice in our institution.

## Results

From 1988 to 2014, we diagnosed 14,816 cases of non-Hodgkin lymphoma, 10958 (73%) were of B-cell histology; and 3822 (26%) were of T-cell histology. In 36 cases, the type of cell was not identified, because the slides were not available for revision, and these cases were not included in this paper.

Table 1, shown the histopathology of the T-cell lymphoma, most cases (40%), were NKTCL, followed by peripheral T-cell non-specified, other T-cell lymphomas were rare.

Table 2 shows the clinical and laboratory characteristics of the NKTCL patients. According the stage, the early stages were more frequent. Also according the clinical risk, the low and intermediate forms evaluated by IPI, and the groups 0 and 1, by Korean prognostic model for nasal NK/T-cell lymphoma were more frequently found.

As expected, patients with advanced stages: III and IV, had poorer prognosis factors, a clinical high risk in both systems and a poor performance status. Curiously, of the advanced stages, only 21 patients (2.7%) were at stage III.

Table 3 shows that, in early stages, radiotherapy attained a complete response (RT) of 73%, of patients, but the relapse, especially outside the radiation site, was frequent. Salvage chemotherapy rescued more patients, but the overall survival (OS) was low when compared to combined therapy. We did not treat advanced stages with radiotherapy alone. The best results were achieved with the use of combined therapy; “sandwich technique” is considered the best combination. Taking into consideration the poor results with chemotherapy alone in early and advanced stages, we did not recommend chemotherapy alone in this type of lymphoma.

Table 4 shows a comparative analysis of patients from different countries.^8^–^18^ NKTCL, nasal type, is a rare disease in Europe and North-America, also in the Latin-American countries, with a high proportion of European-migration, as Chile and Argentina.

## Discussion

In Mexico, NKTCLs represent the 40% of all T-cell lymphomas, and 10% of all malignant lymphomas, diagnosed in our hospital. The International T-cell Project was the first attempt to explore the geographic variations about the T-cell lymphomas; and specifically of NKTCL. These results showed that the NKTCLs represent about 10% of the total of T-cell lymphomas found in North America, Europe, and Asia.^8^ The percentage ranged from 4.3% in Europe to “2.4% in Asia. In the USA, the percentage was 5.1%. Some years, ago, the study was repeated in another countries, with small variations In a subsequent study, also made in the USA, Bellesi et al.^9^ reported that the NKTCLs were the 13% of all 737 cases of T-cell lymphomas, with a percentage ranging from 6% in Europe and 31% in the Middle/Far East. In the USA, the percentage was the 9%. Recently Dubal et al., performed a retrospective analysis of cancer of head and neck in the USA, and found 1382 cases of sinonasal lymphomas, but only 328 (23%) were considered that were NKTCL. Moreover, the analysis for a general population showed that NKTCLs in the USA represent only 0.032/100,00 habitants.^10^ The T-cell Project report that the frequency in European countries, the presence of NKCTLs is rare, 4.3 % of 1314 cases,^8^ and subsequently 6.0% of 737 patients.^9^ Isolated reports from some European countries has been published: Italy: 26 cases;^12^ Portugal: 12 patients, which were analyzed the molecular changes found in these patients.^13^ Marcos-Gragera in a retrospective survival analysis of a different form of lymphoid neoplasms in European Countries reported that NKTCLs were not present.^14^

Laurini at Al. have presented the incidence of NKTCL in Central and South America in the confrontation with the USA.^11^ In this report these neoplasm represent 0% in the USA and the 23 % of all T-cell lymphoma (27 cases in 118 cases of T-cell lymphoma) in Central/South America, respectively 13 %(4/31 cases) in Peru, 4.5% (1/22 cases) in Argentina, 9% (2/18 cases) in Brazil, 22 % (5/22 cases) in Chile, 66% (15/25) in Guatemala, 13% (4/31) in Perù.^11^

Bellesi et al. reported 9 cases (11%) in Chile, and 8 cases Brazil (15%) among 152 cases found in South America.^9^ Gualco et al. reported 122 Brazilian patients, but they only analyzed the presence of the subtypes of EBV virus in patients with NKTCL.^18^ However, these differences could be considered to because they were performed in different populations.

The high rate of frequency of NKTCL in Guatemala, Central America, is also confirmed by the report of Van der Rijn et al..^14^ In the analysis of neoplasms of head and neck, these authors found that 17 cases (88%) were NKTCL, this search was also able to detect that many patients were positive for EBV. Although, nasal NK/T-cell lymphoma shown a higher prevalence of EBV seropositive, the role in the pathogenesis of nasal NKTCL not has been defined. Other forms of the chronic EBV-associated disease are not common in the Mexico. Ortega et al...^16^ reported a Mexican study, analyzed 264 cases of non-Hodgkin’s lymphoma with the nodal and extranodal presentation. In the extranodal presentation, 55 cases, 16 patients were considered lymphoma of the midline or centrofacial with angiocentricity. Thus, we considered that were NKTCLs, even if the molecular profile was the most important task of this research.^16^ Perry et al.^17^ in another report from Guatemala, analyzed 226 patients with non-Hodgkin’s lymphoma, 25 cases were of T-cell origin, and 18 (7.9 % of the total of cases) were NKTCL, no other dates were included.

## Conclusions

The number of NKTCL cases found in Mexico was similar to that found in Guatemala and Peru, and also in China and Korea. Our study suggests that this neoplasm could have a racial basis, but environmental factors also had to be considered.

According our experience^5^ and of others concomitant/sequential chemotherapy and radiotherapy is the standard treatment.^20^ Radiotherapy alone, also for the first stages, is inadequate because of high systemic failure rate.

## Figures and Tables

**Table 1:** T-cell lymphoma. Mexican Population.

	N°	%
All T-cell Lymphoma	3822	
Nasal NK/T-cell lymphoma	1524	40
Peripheral T-cell lymphoma, not otherwise specified	1084	28
Anaplastic large cell lymphoma, ALK +	587	15
Anaplastic large cell lymphoma, ALK −	279	8
Angioimmunoblastic T-cell lymphoma	199	5
Hepato-splenic	60	<1
Subcutaneous panniculitis, like	35	<1
Enterophaty, T-cell lymphoma	29	<1
Adult T-cell leukemia lymphoma	25	<1

**Table 2:** Nasal NK/T-cell lymphoma. Mexican population.

	Stage I-II No (% )	Stage III-IV No (% )
**Number**	1129 (75)	395 (25)
Age (years): median Range	50.1	46.8
	22–64	27–60
Male	601(53)	198 (44)
IPI: Low-Low-intermediate	989 (87)	149 (37)
IPI: High-intermediate/High	140 (12)	246 (62)
Performance status (ECOG) <2	1004 (88)	188 (47)
Performance status (ECOG) >2	125 (11)	207 (52)
Bulky disease ( nasal site) cm.>10	301 (21)	67 (16)
Bone Marrow infiltration	0	127 (36)
Korean Prognostic Classification 0	608 (53)	26 (6)
Korean Prognostic Classification 1	414 (36)	84 (21)
Korean Prognostic Classification 2	89 (8)	110 (27)
Korean Prognostic Classification 3	8 (07)	175 (44)

**Table 3:** The outcome from Nasal NK/T-cell lymphoma in the Mexican population.

	Radiotherapy	Chemotherapy		Combined Therapy	
Stages	I–II	I–II	III–IV	I–II	III–IV
Complete response	73%	45%	NT+	90%	60%
Progression-free survival *	48%	40%	NT	84%	60%
Overall survival *	71%	37%	NT	91%	65%%

+ Not treated,

* Actuarial curves to 10 years.

**Table 4:** Frequency of T-cell lymphoma and NNK subtype by country and region

	Number of Cases	Number %
**Regions and Reports**	T-cell lymphoma	Nasal NK T-cell lymphoma
**North America, Europe, Asia**^8^	1314	10.4
North America		5.1
Europe		4.3
Asia		22.4
**South America, Europe, and Middle/Far East**^9^	737	13
Europe	317	6
Italy		5
United Kingdome		4
Spain, Switzerland, Slovakia		11
USA		9
South-America	152	13
Chile		11
Brazil		15
Argentina, Uruguay		15
Middle/Far East (South Korea; Hong Kong; Israel)	338	31
**Central and South America, North America**^11^	118	23
Argentina		4
Brasil		9
Chile		22
Guatemala		66
Peru		13
North America	38	0
**Europe-North America, Asia**^19^	1153	8
Europe-North America	689	3
Asia Total	464	15
Bangkok, Thailand		34
Hong Kong, China		21
Seoul, Korea		19
Japan Total	306	12
Tokyo, Japan		13
Nagoya, Japan		17
Okayama, Japan		20
Fukuoka, Japan		6
